# Medically uncontrolled conjunctival pyogenic granulomas: correlation between clinical characteristics and histological findings

**DOI:** 10.18632/oncotarget.13961

**Published:** 2016-12-19

**Authors:** Dan Wu, Tingting Qian, Takeshi Nakao, Jianjiang Xu, Zuguo Liu, Xinghuai Sun, Yiwei Chu, Jiaxu Hong

**Affiliations:** ^1^ Department of Ophthalmology and Visual Science and Eye Research Institute, Eye, and ENT Hospital, Shanghai Medical College, Fudan University, Shanghai, China; ^2^ Department of Immunology, Shanghai Medical College, Fudan University, Shanghai, China; ^3^ Massachusetts Eye and Ear Infirmary, Harvard Medical School, Boston, Massachusetts, USA; ^4^ Eye Institute of Xiamen University Fujian Provincial Key Laboratory of Ophthalmology and Visual Science, Xiamen, Fujian, China; ^5^ Eye Center and Department of Ophthalmology, The Second Xiangya Hospital and Eye Hospital, Central South University, Hunan, China; ^6^ Key Laboratory of Myopia, National Health and Family Planning Commission, Shanghai, China

**Keywords:** pyogenic granulomas, clinical characteristic, histology, Pathology Section

## Abstract

**Background:**

Conjunctival pyogenic granulomas are commonly seen after ocular surgeries or at an ocular wound site. The aim of this study is to describe a novel histological classification for medically uncontrolled conjunctival pyogenic granulomas (MUCPG), and to explore whether the diversity in clinical features correlates to different histological subtypes of MUCPG.

**Methods:**

This is an observational cross-section case series. We reviewed 46 consecutive patients with conjunctival pyogenic granulomas who did not respond to topical corticosteroids and underwent surgical excision from January 1, 2006 through December 31, 2015. Clinical features and histological findings were presented and analyzed.

**Results:**

Ocular surgery, accidental injury, and chalazion were the main predisposing causes of MUCPG. The lesions tended to occur unilaterally on the bulbar conjunctiva. Forty patients (87%) presented an enrichment of inflammatory cells and proliferated capillaries in their pathological sections (inflammatory pattern). Six patients (13%) showed relatively few inflammatory cells and capillaries within fibrous stroma (fibrous pattern). Patients with the inflammatory pattern were older (p = 0.025) and tended to be located in bulbar conjunctiva (p = 0.002). The predisposing causes were also different between two histological subtypes (p = 0.007).

**Conclusions:**

We found the correlation between clinical presentation and histological subtypes in patients with MUCPG, indicating this disease may need a new classification scheme.

## INTRODUCTION

Pyogenic granulomas are recognized disorders that can affect ocular mucous membranes [1]. Conjunctival pyogenic granulomas are most commonly seen after surgery for enucleation, pterygium, lacrimal duct obstruction, and strabismus, or at an ocular wound site [1–6]. Previous histologic findings in pyogenic granulomas showed mixed inflammatory cells, blood vessels, and connective tissue [7–9]. Treatment with topical corticosteroids is effective for approximately 90% of patients with conjunctival pyogenic granulomas [3]. For those who do not respond to topical medication, surgical excision is strongly recommended.

So far, very little has been reported about clinical and histological features of medically uncontrolled conjunctival pyogenic granulomas (MUCPG). Ferry systematically described the predisposing causes and clinical presentation of patients with MUCPG in a retrospective pathologic review [1]. Espinoza and Lueder found that patients with MUCPG had shorter courses of onset than those cases sensitive to topical medication treatment [3]. Jorden showed that MUCPG were found to occur at surgical and nonsurgical sites after oculoplastic procedures [9]. Shields et al reported that MUCPG usually presented as a slowly enlarging fleshy vascular mass [10]. The relationship between clinical features and histological findings remains elusive. Therefore, we presented a series of 46 patients with MUCPG from a tertiary eye center and hypothesized that the diversity in clinical features correlates to different histological subtypes of MUCPG.

## RESULTS

A total of 46 cases of MUCPG has been identified in the past 10 years. The mean age was 23.5 ± 20.7 years (range, 2–68), and 36 patients (78.3%) were male, 44 cases were unilateral, and the median duration of the disease was 2.5 months (interquartile range, 1.0–6.0). The affected anatomic sites included bulbar conjunctiva (n = 37, 80.4%) and tarsal conjunctiva (n = 9, 19.6%). The mean lesion diameter was 7.2 ± 3.5 mm (range, 3–15 mm). As shown in Table 1, the specific predisposing causes included ocular surgery (n = 22, 47.8%), injury (n = 11, 23.9%), chalazion (n = 8, 17.4%), and Stevens-Johnson syndrome (n = 2, 4.3%), and the remaining causes were not determined (n = 3, 6.5%).

**Table 1:** Demographic data of 46 consecutive patients with medically uncontrolled conjunctival pyogenic granulomas

	Total (n = 46)	Inflammatory pattern (n = 40)	Fibrous pattern (n = 6)	p value*†
Age (years)	23.5± 20.7 (range, 2-68)	25.8 ± 21.2 (range, 2-68)	8.7±7.4 (range, 3-22)	0.025
Sex				0.327
Male	36 (78.3%)	31 (77.5%)	5 (83.3%)	
Female	10 (21.3%)	9 (22.5%)	1 (16.7%)	
Laterality				0.634
Right eye	21 (45.7%)	19 (47.5%)	2 (33.3%)	
Left eye	23 (50.0%)	19 (47.5%)	4 (66.7%)	
Bilateral	2 (4.3%)	2 (5.0%)	0	
Time since onset (months)	Median 2.5 (interquartile range, 1.0-6.0)	Median 3.0 (interquartile range, 1.0-6.0)	Median 1.5 (interquartile range, 0.9-4.5)	0.170
Predisposing cause				0.007
Ocular surgery	22 (47.8%)	21 (52.5%)	1 (16.7%)	
Enucleation	14	13	1	
Pterygium surgery	2	2	0	
Lacrimal intubation	3	3	0	
Strabismus surgery	3	3	0	
Injury	11 (23.9%)	11 (27.5%)	0	
Burn	8	8	0	
Trauma	3	3	0	
Chalazion	8 (17.4%)	4 (10.0%)	4 (66.6%)	
Steven-Johnson syndrome	2 (4.3%)	2 (5.0%)	0	
Undetermined	3 (6.5%)	2 (5.0%)	1 (16.7%)	
Location				0.002
Bulbar conjunctiva	37 (80.4%)	35 (87.5%)	2 (33.3%)	
Tarsal conjunctiva	9 (19.6%)	5 (12.5%)	4 (66.7%)	
Lesion Size	7.2±3.5 (range, 3-15)	7.3±3.5 (range, 3-15)	6.0±3.0 (range, 3-10)	0.345

* A chi-square test or the Mann-Whitney U test was performed between two groups.

†P < 0.05 considered statically significant.

**Figure 1:**
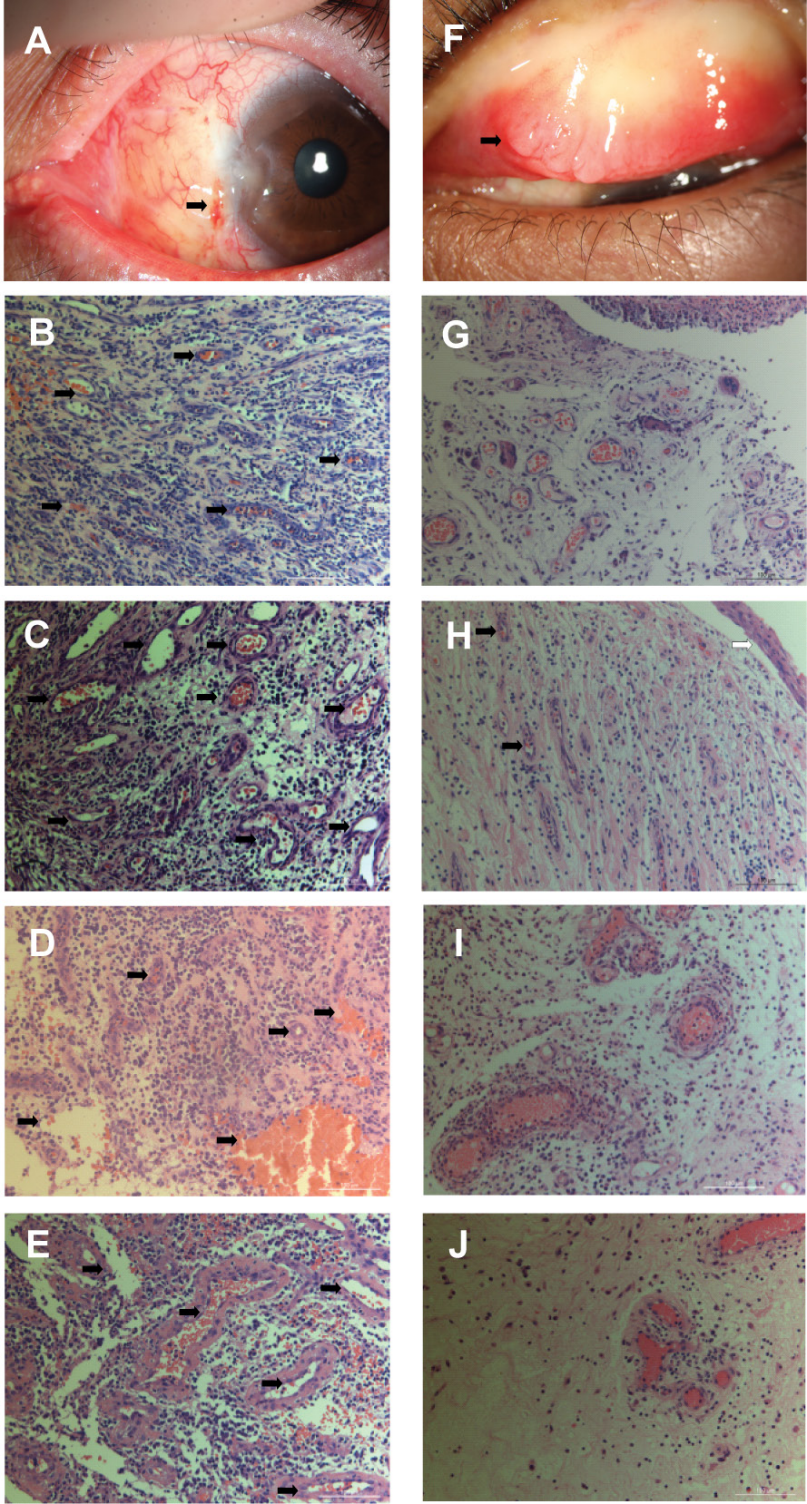
Clinical and histological appearance of medically uncontrolled conjunctival pyogenic granulomas **A.** The clinical image shows a granulation tissue at the site of a suture after pterygium surgery (black arrow). **B.~E.** The inflammatory pattern in pathological sections shows prominent proliferation of capillaries (black arrow) with an associated intense infiltration of inflammatory cells (65532 ± 8563 cells/cm2), mainly including lymphocytes, plasma cells, and neutrophils. **F.** The clinical image shows a granulation tissue at the site of a chronic chalazion (black arrow). **G.-J.** The fibrous pattern is characterized by loose fibrous stroma with fewer inflammatory cells and capillaries (34628 ± 10563 cells/cm2, P < 0.001). For example, as shown in , an intact, attenuated epithelium (white arrow) covered a lesion which had a loose, edematous stroma with various degree of inflammatory reaction and blood-filled capillaries (black arrow) lined by vascular endothelial cells. (stain, hematoxylin–eosin; original magnification, ×20)

Based on the histological features, we divided patients into two groups: the inflammatory pattern group (seen with many inflammatory cells and proliferated capillaries) and the fibrous pattern group (seen with fibrous stroma and relatively few inflammatory cells and capillaries), which corresponded to the variable components within the specimens (Figure 1). Histological pictures showed that the inflammatory pattern of MUCPG had prominent proliferation of capillaries with an associated intense infiltration of inflammatory cells, mainly including lymphocytes, plasma cells, and neutrophils (Figure 1A–1E). Relatively, the fibrous pattern was characterized by loose fibrous stroma with a few inflammatory cells and capillaries (Figure 1F-1J).

In addition, as shown in Table 1, the relationship between clinical features and histological findings in patients with MUCPG was explored. Both groups were comparable in terms of sex (p = 0.195), duration of the disease (p = 0.170), the laterality of eyes affected (p = 0.634), or the lesion size (p = 0.345) (Table 1). However, our data found that patients with the inflammatory pattern were older than those with the fibrous pattern (p = 0.025). Inflammatory MUCPG tended to be located in bulbar conjunctiva, whereas fibrous MUCPG were located in tarsal conjunctiva (p = 0.002). The predisposing causes were also different between two histological subtypes (p = 0.007).

## DISCUSSION

In the current study, we found that patients with an inflammatory patterns were older, more likely affected in the bulbar conjunctiva, and secondary to ocular surgery or accidental injury, whereas patients with a fibrous pattern seemed to be younger, more likely to be affected in the tarsal conjunctiva, and related to chalazion.

Clinical features of patients with MUCPG in our study were different from previous reports. In an early case series of 100 surgically treated ocular pyogenic granulomas, Ferry reported that chalazion (42%) and ocular surgery (40%) were the main predisposing causes [1]. He also noted that conjunctival pyogenic granulomas after surgical treatments arose mostly in scleral buckling, strabismus, pterygium, pinguecula surgeries, and plastic surgery of the eyelids. In a case series of Jorden et al., enucleation and lacrimal surgeries dominated the type of surgeries preceding pyogenic granulomas formation [9]. Our study showed that ocular surgery and accidental injury play important roles in the pathogenesis of MUCPG.

The etiology of conjunctival pyogenic granulomas remains obscure. Previously, the proliferation of capillaries and the infiltration of inflammatory cells have been linked to cytokines, including vascular endothelial growth factor, fibroblast growth factor, and proinflammatory factors [9, 11, 12]. The relationship between clinical and histological data on the inflammatory pattern partially confirmed a previous hypothesis, which proposed that conjunctival pyogenic granuloma formation results from an angiogenic imbalance during wound healing [9]. As shown in our results, in most of patients with this pattern, it occurred after ocular surgery and accidental injury. This may be explained by the fact that the persistent mechanical (integrated orbital implant and silicone tube) or chemical irritation (organic burn) can lead to an imbalance of mediators released, resulting in excessive vasoproliferation. Conversely, the majority of MUCPG with a fibrous pattern occurred in patients with chronic chalazion (66.6%), whose inflammatory reaction seems to be self-limited during the wound-repair process. MUCPGs with inflammatory patterns largely occurred in the bulbar conjunctiva, and more than half of MUCPGs with fibrous patterns occurred in the tarsal conjunctiva. These results are reasonable because MUCPGs with fibrous patterns were mostly chronic chalazions. One may suspect that the fibrous character of MUCPG may be related to a long-term onset time of wound healing. However, our data that the histological pattern was not related to duration of the disease did not support this speculation.

The cross-section design of this single-center study poses some inherent limitations. The data were obtained over a 10-year period based on the pathological database, entailing incomplete medical records of patients responsive to topical medication without biopsy information. Furthermore, we did not have the data on the follow-up and lacked of quantitative analysis for inflammatory cell density and capillaries density in pathological sections. Finally, the relatively small size of samples, especially the low number of fibrous pyogenic granulomas compared to the inflammatory category, may skew the correlation analysis. Our preliminary data still require independent investigation with a reasonable number of patients to verify.

In conclusion, the results of this study confirm the correlation between clinical features and histological findings in MUCPG. Our findings support the establishment of a clinical classification scheme of MUCPG based on histological subtypes, which may help clinician evaluate, diagnosis, and treat patients with conjunctival pyogenic granulomas before the histological results are available.

## MATERIALS AND METHODS

The current study was approved by the institutional review board of the Shanghai Eye, Ear, Nose, and Throat Hospital, Fudan University between January 2006 and December 2015. The principles in the Declaration of Helsinki were followed. By using the database of the Pathology Service at our institute, we retrospectively reviewed the medical records of 46 consecutive patients coded as having conjunctival pyogenic granulomas and reported to be unresponsive to topical medications. Patients with presumed conjunctival pyogenic granulomas but without histological evidence were excluded in our study.

For data collection, each case was evaluated for patient age, sex, laterality, duration of disease, predisposing causes, anatomic locations, and histological reports (including the size of the lesion and the pathological description). Information on the size of the lesion and histological features was obtained from the histological reports.

We used the Mann-Whitney U and Chi-square tests to compare the differences between the two studied groups. A two-sided p value of less than 0.05 was considered statistically significant. Data were analyzed with the statistical software package SPSS 22.0 for Windows (IBM; Chicago, Ill.).
